# Remote Effects of Anæsthesia on the System

**Published:** 1857-04

**Authors:** 


### ARTICLE XII.

### *Remote Effects of Anæsthesia on the System.*

Dr. Frederick D. Lente records (*New York Journ. Med.* Nov. 1856) the three following cases, in which anæsthetics appear to have been productive of serious ill consequences, and which have occurred in his practice within the last five years:

*Case* 1.—In the summer of 1853, assisted by Dr. Leroy, formerly resident surgeon of the New York Hospital, I operated on a boy in apparent good health, eight years old, for contraction of the index and middle fingers of the right hand, the result of the cicatrization of a bone some years previously. As the case required a careful and somewhat protracted dissection of flaps into the palm of the hand, the patient was subjected to the influence of sulphuric ether, administered by Dr. Leroy, on a sponge in the usual way. Nothing remarkable occurred either during the administration of the anæsthetic or during the operation, and but a moderate quantity of blood was lost. The patient soon recovered consciousness, but in a short time he became very feeble, and soon commenced vomiting, although no food had been allowed for seven hours previous to the operation. The pulse commenced sinking rapidly, consciousness being unimpaired. Frictions were at once resorted to, and stimulants attempted, but were immediately rejected by the stomach. The prostration soon became extreme, and dissolution appeared imminent both to Dr. Leroy and myself. Brandy was freely administered by enema, and retained, and in the course of an hour or two, reaction slowly commenced, but it was not until several hours had elapsed that it was considered safe to dress the wounds, so slowly did the patient recover from the prostration.

*Case* 2.—This patient, a young man in ordinary health, not robust, æt. about twenty-five, of nervous temperament, wished to have a large number of decayed teeth and fangs of teeth removed. At the request of the dentist who was to operate, I administered sulphuric ether, patient sitting upright in the operating chair, a necessary position during such an operation. The patient had previously been considerably frightened both at the idea of the operation, and of the anæsthetic, although unwilling to undergo the suffering without it; he had accordingly primed himself pretty thoroughly with brandy, but was in nowise intoxicated. Nothing unusual occurred during the administration of the ether, and anæsthesia was induced without difficulty. Six stumps were rapidly and skillfully extracted, say within three minutes, perhaps within two. The patient then showed some signs of returning consciousness, and more ether was administered; anæsthesia was soon re-established, and six more teeth were, with equal rapidity, extracted. The anæsthesia was very complete, but there was no unusual difficulty in recovering the patient, and he was soon able to walk home. A week or two after this, he applied to me, complaining of debility, pain about the head, and dizziness, a disposition to faint and fall down, and various nervous symptoms, which, he said, had troubled him ever since the operation. He was very low spirited and fearful of some serious disease. He, of course, attributed all this to the ether. I endeavored to divert his mind from this idea, and prescribed change of air and tonics. He went away, but returned within a few weeks not much better. Subsequently he improved, and after a couple of months longer was much better, though still rather nervous and desponding. He afterwards went to the city to reside, and since that time I have not seen him.

*Case* 3.—W. M., a young gentleman, about 30 years old, in robust health, of temperate habits, was attacked with ulceration of the soft parts of the mouth from pressure of a crowded wisdom tooth; the pain was very severe, causing loss of rest and food. I advised the extraction of the tooth, but the dentist to whom he applied merely cut away the overhanging edges of the ulcer; the inflammation increased and extended to such a degree as to produce almost complete closure of the jaws, with inability to open them. It was absolutely necessary now that the tooth should be extracted as the only means of arresting the inflammation, and it was, therefore, proposed to etherize the patient in order to allow the jaws to be forced open sufficiently to admit the introduction of a forceps. Sulphuric ether was accordingly administered; the patient came rapidly under its influence, scarcely requiring an ounce and a half, though not entirely unconscious; the jaw was forced open with but little difficulty, and the tooth rapidly extracted by the dentist in attendance. The patient soon recovered, but seemed a little nervous and considerably excited, but expressed himself as entirely relieved from the severe pain he had been suffering. He was advised to go home and lie down for a few hours. He walked home, about a quarter of a mile or more, and followed my advice; but in the afternoon complained that the ether was still in his lungs, and sought to get rid of it by riding and walking. In the evening he was at the house of a friend in gay society, and seemed to enjoy himself, still, however, occasionally complaining of some difficulty about his chest, when, all at once, he fell from his chair, exhibited great restlessness, tossing about of the arms and legs, with great difficulty of breathing, but no loss of consciousness, declaring all the time that he could not get his breath for the ether, and that he should die; his hands and feet were said to be cold. Before I reached him, various restoratives had been applied, and he had been almost drowned by the assiduous application of hot water. It was evident at once that it was a case of violent hysterics, unusually well marked in a male. Patient at times would laugh and joke, then express fears of impending suffocation, with jactitation, declaring that as vapor of ether was heavier than air, he ought to be held up and allow it to run out of his lungs. As he was rather weighty to allow of convenient inversion, his request was not granted. Large doses of morphine were administered, but had no effect; it was only after several hours that he could be quieted. The next day he was able to be up, but complained of weakness and a disposition to faint on the slightest attempt to walk, also of some difficulty of breathing. This continued for some days, but finally disappeared, and, within ten days, he was apparently in his usual condition. Patient had never previously exhibited any tendency to hysteria."

